# The practice of dealing with existential questions in long-term elderly care

**DOI:** 10.1080/17482631.2018.1508197

**Published:** 2018-08-20

**Authors:** Wander van der Vaart, Rosanna van Oudenaarden

**Affiliations:** Department of Research Methodology, University of Humanistic Studies, Utrecht, The Netherlands

**Keywords:** Elderly care, existential questions, meaning in life, spiritual counsellor, care home, care practice, professional quality framework

## Abstract

**Purpose:** Existential questions concerning life and death or meaning in life are very salient for many older people receiving long-term care. However, little is known about how long-term care organizations deal with existential issues. This study describes the practice in two long-term care organizations that took part in a Dutch pilot programme on existential questions and formulates recommendations for building a professional quality framework. **Method:** Starting from theoretical notions on ultimate concerns, life events and meaning in life, organization policy and care practices were explored. Existing documents, in-depth interviews and participatory observations were used as data sources. **Results:** The outcomes indicate that the long-term care organizations had little active policy on dealing with existential questions; also, personnel had few specific tools except listening closely. Central requirements for dealing with existential questions appeared to involve organizational framework conditions like the availability of spiritual counsellors, having facilities in the building, and clear roles for various actors. Moreover, social-communicative competence building for care personnel was found to be an important demand as well as an organization-wide attitude that puts residents at centre stage. **Conclusions:** A professional quality framework requires a “tiered system” that differentiates organizational roles in dealing with existential questions.

## 1. Existential questions and meaning in life in elderly care

### 1.1. The relevance of dealing with existential questions

For a number of years, existential questions have been increasingly recognized as a central issue in elderly care and, as such, have been the topic of a growing number of studies (e.g., Gijsberts, Van der Steen, Muller, Hertogh, & Deliens, 2013; Kane et al., 2003; Lee, 2002; Morgan & Farsides, 2009; Nolan, 2011; Orton, 2008; Paley, 2008; Sussman & Dupuis, 2014). Existential questions refer to fundamental issues of life: issues of life and death, our place in this world, what it means to be human, what we take for true and valuable or what makes life worth living (Frankl, 1959; Yalom, 1998). In particular, for older people receiving long-term care, existential questions are a salient issue: they are in their final years of life, have lost much of their independency and have to deal with physical, mental and social adversities (Hertogh, 2006, 2013). Usually, existential questions are not so outstanding for people until serious life events happen, or when life circumstances force people to reorient themselves (Cohler & Hostetler, 2003). This process of (re)orientation in life is often understood in terms of a search for meaning in life; existential questions, then, refer to the questions that drive this (re)orientation on meaning in life (Frankl, 1959; King & Hicks, 2009).

The importance of dealing well with existential questions is also emphasized by emerging scientific evidence suggesting that experiencing life as meaningful is accompanied by (and possibly promotes) better physical health, in the form of lower stress hormones, lower inflammatory response, better immune function, lower cardiovascular risk, slower neural response to aversive stimuli, and lower mortality (Krause, 2009, 2012; Ryff, 2012). Research has also shown that while health is considered important by most elderly people, this applies less to ill than to healthy elderly people: aspects of life that become more important for ill older adults are mental health, housing, family and friends, meaningful time expenditure, and religion. In a qualitative study, Helvik, Cabral Iversen, Steiring, and Hallberg (2011) added that issues of meaning in life play a role in maintaining well-being in older people with a chronic illness. In particular, in the case of deteriorating health, creating meaning in everyday life was an adjustment strategy to gain or keep control and balance in life.

In spite of its practical importance, little research is available regarding the role of meaning in life in long-term care practices. It can be expected, though, that especially for older people with chronic conditions, as in long-term care institutions, issues of meaning in life are highly significant (Deeg, 2007; Krause, 2012). Given the societal relevance of this issue, the Dutch National Health Care Institute commissioned the development of a national quality framework titled “Dealing with Existential Questions in Long-term Care for Elderly People” (In Dutch only: https://www.zorginzicht.nl/bibliotheek/levensvraag/Paginas/Home.aspx). The quality framework aimed to set out norms, guidelines and suggestions for Dutch long-term care organizations with regard to good elderly care. The present qualitative study was designed to inform the development of this national quality framework and aimed to generate knowledge that could support long-term care institutions to deal with the existential question of their older residents. To that aim it involved collaboration with the Dutch Expertise Network Meaning in Life and Elderly and its constituting branch institutions and related long-term care organizations. This study approach acknowledges, as put forward by Todres, Galvin and Holloway (2009), that qualitative research can support a more humanizing emphasis for care, not only by showing people’s own perspectives, but also through providing more general insights in human and ethical dimensions that may guide practice and policy.

The research question that is addressed consists of two parts: first, it examines how long-term elderly care organizations (their managers, staff and care personnel) deal with the existential questions of elderly residents (a), and next, what demands personnel and residents put forward for dealing with existential questions (b). Based on the outcomes, we discuss what lessons can be drawn for designing a professional quality framework for this care domain.

### 1.2. Life events and ultimate concerns

While existential questions drive the search for meaning in life, “connection” is often seen as a keyword to define meaning in life (Baumeister & Vohs, 2005; Cohler & Hostetler, 2003): one’s own experiences and one’s whole life are placed within a wider network of connected meanings. In the literature, several dimensions of the concept of meaning in life are distinguished. Baumeister (1991, pp. 29–57) presented his well-known four different needs for meaning: (1) need for purpose; (2) need for moral worth (moral justification); (3) need for self-worth and control; and (4) need for competence, or efficacy. Reviewing the literature, Derkx (2013) added three needs to Baumeister’s classification: (1) the need for comprehensibility, (2) the need for connectedness with others, and (3) the need for transcendence (going beyond the ordinary). Derkx concluded that these seven dimensions—which partly interrelate and overlap—largely cover the conceptual space mapped out in existing studies on meaning in life. Research indicates that older people may have other strategies for finding meaning than the general population (Carstensen, 2006; Westerhof, 2010). However, no reason has yet been found to expect that meaning in life as such would need a different conceptualization for older people.

Given that connection is seen as vital to meaning in life (Baumeister & Vohs, 2005; Cohler & Hostetler, 2003), it is not surprising to find that *life events* play a central role across theoretical meaning in life approaches. Life stories that build on connections and associations between life events are commonly seen as central to detecting and (re)constructing meaning in life (Baumeister, 1991; Cohler, 1993; Cohler & Hostetler, 2003; Van Praag, 1978). Life events play a dual role: on the one hand they challenge existing forms of meaning, and on the other hand they may function as anchors for the (re)construction of meanings (Baumeister & Vohs, 2005; King & Hicks, 2009). For older people receiving long-term care, one may think of life events such as leaving one’s home and moving into a care residence, the death of family members and co-residents, or illness, but also positive events like the birth of a grandchild, marriages and birthdays.

In a highly pertinent way, relationships between major life events and existential questions come to the fore in “ultimate concerns”, strong experiences of deprivation or fulfilment of meaning, as identified by Yalom (1980, 1998). Yalom distinguished four types of ultimate concerns that affect every individual, being related to four major life events or experiences: death, freedom, existential isolation or loneliness, and (the fear of) meaninglessness. The ultimate concerns refer in their own way to existential conflicts (Yalom, 1998):

- For “death”, this is the tension between being aware of the inevitability of death and the desire to remain existent, to continue life.
- Regarding “freedom”, it refers to the human desire for external structure, while our culture assumes that, in the end, people alone are responsible for their own choices and behaviour (cf. Baars, 2012; Gilleard & Higgs, 2000, 2005).
- For “isolation”, the existential conflict consists of the tension between the human need for relationships and being a part of a bigger whole and the awareness of a fundamental isolation from others and the world.
- Finally, for “meaninglessness”, the dilemma is represented by people in need of meaning, being in a world that basically has no meaning at all.
- Since not only the negative side of the “ultimate concerns” may be of importance to meaning in life, their positive counterparts—life (birth), dependency (structure), connectedness and meaningfulness—are also explicitly explored in our empirical study.

The experience that the giving of meaning stagnates, that people encounter a lack of coherence and orientation, can be understood as existential distress (Frankl, 1959; Yalom, 1980). Conversely, we might speak of existential satisfaction when people have a strong experience of meaning and coherence. Following the seven meaning in life dimensions identified by Derkx (2013), existential distress may arise if one or more of the seven needs are not satisfied. From the perspective of Yalom’s “ultimate concerns” (Yalom, 1998), existential distress occurs when people are faced with one of the existential themes (related to negative or positive events) and get caught up in existential conflicts. In both approaches the positive counterparts can represent existential satisfaction.

In our study we draw on both perspectives. We are using the four ultimate concerns to explore the role of existential questions in elderly care, and make them tangible by focusing on actual negative and positive life events. The aforementioned seven dimensions of “meaning in life” allow us to take a more detailed look at possible relationships between aspects of meaning in life and the way the selected care organizations work.

## 2. Methods

A multiple-case study (Yin, 2014) was designed involving two purposefully selected organizations for long-term elderly care. The two selected long-term care organizations took part in a pilot programme of the Dutch Expertise Network Meaning in Life and Elderly, meaning that both organizations had an active policy on dealing with existential questions. The latter is rather unique and this made it exceptionally suitable for the present study that aims to explore how care organizations deal with existential questions. Organization A’s policy vision was to sustain clients as unique personalities with their own future and life history and it aspired to creating a community dedicated to well-being. As a consequence, its policy aimed to steer by values instead of steering by protocols. Organization B aspired to tailor-made care that stimulates clients’ talents and qualities and supports them to live an autonomous life. As a consequence, Organization B installed a quality monitoring system that focused on client outcomes instead of on procedures. Both organizations thus had a policy of trying to stimulate tailor-made care that serves well-being and meaning for each specific client.

In addition, both organizations fulfilled our criterion of being a large-scale care institution; this was a necessity since our research question adresses a broad range of issues in the care organization (related to both policy and care practice). Both long-term care organizations were Protestant Christian institutions, with multiple locations, encompassing an elderly nursing home, senior residential homes and home care.

This study encompassed the nursing homes of both organizations, each giving a home to around 140 residents. Research focused on three levels of the organization: (a) management and policy staff, (b) health care staff, and (c) elderly residents and their social network.

Data were gathered through (policy) documents, in-depth interviews with older residents, and participant observation in both organizations. Since both organizations participated in the aforementioned pilot programme, a substantive amount of existing documentation was available for the current study. By means of participant observations and in-depth interviews we added data on the resident’s own perspective, as well as on how staff meetings addressed existential issues and related policies. We thus used four different types of data collection:

1. Desk research regarding documents gathered by participants in the pilot programme and documents from the two long-term care organizations:
  - Organization policy documents and year reports;
  - Existing accounts of meetings and/or internal research, and accounts drawn up by the researchers of attended organization meetings;
  - Existing documents on the “pilot programme” policy and accounts of programme meetings attended by the researchers.
2. Participant observations of five professional meetings: staff meetings in both organizations and board meetings in the context of the pilot programme;
3. In-depth interviews with six selected older residents from the long-term care organizations;
4. Participant observations of daily activities in the two long-term care organizations, involving both residents and staff: about 8 hours of participant observation, including around 16 informal interviews in total.

The interviews and participant observations were conducted by two qualified research assistants. Both were trained to perform qualitative research and were skilled in qualitative interviewing. Per organization, the same research assistant performed the interviews and observations. Fieldwork was organized with the help of spiritual counsellors in the two nursing homes.

Per long-term care organization, three residents were selected for in-depth interviews. They were chosen in consultation with the spiritual counsellor of the nursing home and judged to be informative, open to being interviewed and able to express themselves sufficiently. All interviewees were women between the ages of 85 and 95 years old; five of them lived alone, one lived together with her husband. Some were physically more constrained or less active than others, but all were in relatively good physical and mental health.

The interviews were semi-structured by using a topic list (Boeije, 2010; Rubin & Rubin, 2016) that was derived from our theoretical framework on existential questions, meaning in life, ultimate concerns and life events. The core of the interview consisted of exploring what happened when the resident was faced with existential questions (positive and negative concerns) and/or when positive and negative life events occurred: where the resident could find support, what happened then, and what the resident wished had happened. Based on the topics, open questions were asked and keywords used to probe further. The interviews were held without bystanders in the residents’ private living room and took 30–60 minutes. The interviewer took notes during the interview and elaborated them immediately afterwards.

The participant observations and informal interviews in both organizations focused on behaviour and interactions as possibly related to the interview themes (Coffey & Atkinson, 1996; Mason, 2012). Observations included environmental features (lounge corners, use of colour, etc.) that might affect atmosphere and behaviour. Conversations between residents and between residents and personnel were observed and the researcher actively initiated informal conversations lasting between a few and 10 minutes.

The goal of the participant observations was to derive clues for ways in which existential questions were attended to. The research assistants went around for several hours visiting various floors at random and observing a diversity of locations. They joined get-togethers organized by volunteers (“coffee time with the neighbours”), visited residents in their own room to include people who did not participate easily in activities, visited the restaurant during staff lunch break, etc. Similar to the in-depth interviews, the observations and informal interviews were elaborated on paper by the research assistant during and after the participant observations.

The theoretical notions regarding existential questions, meaning in life, “ultimate concerns” and life events were used as sensitizing concepts to explore the data (cf. Coffey & Atkinson, 1996; Mason, 2012). The researchers and one of the research assistants coded the documents and accounts using a code scheme that represented these theoretical notions and the topic list. These deductive codes were complemented with codes inductively derived when new themes appeared in the interview and observation accounts. The analysis of both the documents and interview accounts then focused on how long-term care organizations and their personnel dealt with existential questions and what kind of requirements were mentioned in relation to that. Data from the two organizations are taken together as forming one data set, yet differences between both were scrutinized during analysis and are shown when relevant. In Section 3: Results, outcomes of the different data collection methods (documents, observations, interviews) are combined, when available, in each of the presented themes. Since the interviews were conducted with elderly only and focused on existential questions, they primarily resulted in data on spiritual care and less in data on other topics. The outcome sources are mentioned within the descriptions of each theme.

## 3. Results

### 3.1. The practice of dealing with existential questions

The first part of our research question examines how long-term elderly care organizations (managers, staff and care personnel) deal with existential questions. The results are presented in four themes that stood out most: “organization policy and mission”, “daily care activities”, “attention for spiritual care” and “taking care of the living environment”.

#### 3.1.1. Organization policy and mission

Although both long-term care organizations took part as pilot organizations in the “pilot programme on meaning in life”, until then the concept of “meaning in life” did not occur in their policy documents and internal accounts. Also, all these organization documents of both pilot organizations (see Section 2: Methods) did not reveal much information on structural plans with respect to dealing with existential questions (like employment of spiritual counsellors, training programmes, financial resources, facilities, volunteer policy). In addition, the participant observations showed that not all care personnel and residents were familiar with the pilot programme on existential questions.

The matter of world view is an important policy goal for both organizations, though, as is illustrated by the fact that both organizations explicitly name their Protestant Christian identity in their mission statement. Also, both organizations do attach importance to a number of matters that may be seen as “preconditions” for paying attention to existential questions. To keep track of its care quality, Organization B employs a monitoring system in which “mental well-being” features as an important theme. It remains unclear, however, to what extent this quality system actually addresses existential questions. At Organization A, residents’ *well-being* is a key concept that is seen not as a separate aspect of care, but as an integral part of it. The care home is seen as a residential community, in which existential questions and the meaning of life also play a role. This organization sets great store by the quality of the care personnel, specifically regarding their ability to establish contact and to engage the residents in conversation and to give them the opportunity to express whatever concerns them.

#### 3.1.2. Daily care activities

Regarding the care personnel, the documents show for both organizations that personnel recognize existential questions when experienced by residents and by themselves, and that they attempt to pay attention to this. This is not always found easy to do, and some express the wish for more support in this matter. Some personnel appear better equipped to deal with existential questions than others, and residents determine for themselves with whom they feel safe enough to raise such questions. Also, during participant observation the volunteers in both organizations indicated that the ability of personnel to deal with existential questions varies from person to person: some are very tactful, while others show little sensitivity or interest. As the documents of both orga-nizations (see Section 2: Methods) indicate, when existential questions occur, personnel mainly try to show personal attention and to free up time to talk about them. Sometimes they refer the resident to a spiritual counsellor or another expert (e.g., the mental health care staff). Attempts are also made to create the right conditions in other ways, such as gathering enough background knowledge about the residents (personal history, interests, etc.).

#### 3.1.3. Attention for spiritual care

The interviews with residents add that attention for spiritual care is not always reflected in daily care activities. Residents in both organizations indicate that the care personnel generally do try to find the time for personal conversation, but that having attention for what is really on one’s mind is not a matter of course.

As one resident puts it: “There is attention for physical well-being, but not for spiritual well-being; the care personnel doesn’t have enough room for that” (R1, A). Quotations of interviewees refer to respondent number (like R1) and health care organization A or B.

A lack of time and expertise are identified as important limitations. For instance, one resident said that having attention for existential questions requires an expertise that is more fitting for a spiritual counsellor than for care personnel. Staff members do enquire after the respondent’s well-being, but do not probe further. The contact with personnel and volunteers remains superficial and functional, due to a lack of time:

“After you [research assistant] leave, I won’t see anyone for the rest of the afternoon, until they come with tea and a sandwich, and then I won’t see anyone until 9.30 pm again” (R3, B).

Also, there is too much turnover among the personnel to build a deeper bond with them. However, residents also indicate a varying need for attention for existential questions: some have a stronger need for deeper contact than others. This depends both on personal need and on the availability of a social network. It is also the resident’s own responsibility to express his or her needs; but putting too much emphasis on “resident-centredness” can mean placing too much responsibility on the part of the resident:

“The personnel always say: don’t tell us to come when it suits us, we will come when it suits you, you’re the boss here” (R2, B).

Additionally, many residents do not take the initiative to explicitly address the experience of positive or negative forms of meaning (one resident explained that “we lack the language”), and it is much appreciated when personnel take the initiative to broach the subject. One resident reported being very happy with the spiritual counsellor’s discussion group, as this provides the opportunity to talk about existential questions. The spiritual counsellor is not always well known among the residents, though. It even emerged both in the interviews and during participant observations that not all residents and care personnel are aware of the availability of spiritual counsellors.

The respondents at both organizations indicate that they appreciate the normal social interaction, both individually and in the form of communal activities, and provided both by personnel and by volunteers. Opinions are divided as concerns the personnel’s knowledge of important events in the residents’ own lives. Some residents say that the care personnel are not aware of important events, such as a death in the family:

“They know the date of your birthday, but that’s about it” (R1, B).

Others raise the question to what extent the personnel should enquire more and/or what their own role can be: “It’s also your own responsibility to make the personnel aware of important things” (R3, A).

One respondent said that the personnel enquire every day how she is doing (following the death of her partner). Another respondent expressed a strong appreciation for the regular attention given by the spiritual counsellor. The respondents at Organization B say that special events are explicitly marked (for instance, with a birthday cake, or by addressing it during the end-of-week assembly).

#### 3.1.4. Taking care of the living environment

In both organizations the atmosphere in the buildings has aspects that may be of importance for dealing with existential questions. Observations in the buildings demonstrated that comfortable lounge corners can be found on every floor and the atmosphere is generally homely, although the hallways tend to be rather bare. Personnel seem continually busy, though, leaving little opportunity for impromptu conversations. Residents therefore seek out other people to interact with in the communal spaces; for instance, by the coffee machine or in the library. The interviewees confirm this picture: they say that attention for how one is truly doing is not a matter of course. Personnel are generally busy; many of the interviewees say that they would appreciate more personal attention and that things can be a bit lonely at times. One or two say they have close contact with family members and therefore have less need for attention on the part of personnel. The buildings do not have designated contemplation rooms. One striking aspect of Organization B’s building is that it directly faces a garage for funeral cars, so that some residents can see these cars coming and going all day long. Some of them find this quite confronting and unsettling:

“Every day you see those funeral cars go out, I find that a bit scary” (R3, B).

#### 3.1.5. Conclusions

Several main themes emerged on how the long-term care organizations and their personnel dealt with existential questions. The management of both care organizations attaches value to attention for existential questions. This is borne out by the fact that both organizations chose to participate in the pilot programme. Existential questions are made a policy point at times, but this remains very limited. At both organizations, the care personnel mainly cope with existential questions by listening. Documentation and interviews with residents do show that the personnel are very busy and not always competent to deal with existential questions among residents. Not everyone is familiar with the spiritual counsellor. The overall picture is that both the organization and the personnel try to deal with existential questions as best as they can, but that neither organization nor the personnel are fully prepared for this task. The organization’s approach is not always explicit or structural and the personnel apply a limited repertoire of competences in dealing with existential questions. The various sources—the documents, the interviews, the participant observations—complement each other and confirm the picture that arises in these conclusions.

### 3.2. Research question part 2: demands for dealing with existential questions

The results regarding the second part of the research question, on demands that personnel and residents put forward for dealing with existential questions, clustered around two main issues: “organizational and personnel requirements” and “personal communication and relational requirements”.

#### 3.2.1. Organizational and personnel requirements

Documents from the pilot programme—on meetings, internal research and programme policy—reveal organizational requirements and competences of staff that, according to the organization and personnel themselves, are needed for dealing with existential questions. To start with, the documents showed that both organizations—though participating in the pilot programme—seemed unable to explain clearly to the personnel what they mean exactly by existential questions. Discussions on this topic mainly relied on examples with the goal of eliciting recognition. It seems that the personnel are expected to show a certain attitude or sensitivity in this respect. But, as residents stated in in-depth interviews and during participant observations, significant differences among care personnel exist in their ability to deal with residents’ existential questions and that training and support are of great importance. Also, residents wished that attention to existential questions would be offered to them more actively and explicitly by the organization. During participant observations, residents pointed out in addition that organization and personnel should also maintain a focus on the quality of facilities and the (social) possibilities offered by the building.

A recurrent theme is an apparent need for more clarity on the role of various actors. The documents showed that—although the pilot was to be implemented across the organization—no distinction was made between skills for the different functions within the organizations. In regard to that, interviews and participant observations indicated that personnel and volunteers needed more clarity about the roles of different actors. There seems to be some uncertainty in what to expect from whom. Also, a need for experts was expressed, such as spiritual counsellors to whom residents can be referred. Interviewed residents stated that in their opinion, giving support for existential questions is part of the spiritual counsellor’s competence and not that of care personnel or other staff members. But they also find it helpful that personnel and residents are aware of each other’s faith or world view. Uncertainty also applies to what they may expect from volunteers, as well as to the difference between the role of the pastor and that of the spiritual counsellor. Expanding on the spiritual counsellor, residents feel that the spiritual counsellor should fulfil an active role. They added—both in the in-depth interviews and the participant observations—that it is important that he/she personally takes the initiative to pay residents a regular visit or to invite them to a discussion group. This is because residents often find it difficult to broach the subject, they often lack “the language” to address the matter, and they need someone to support this. In this line one respondent illustrates the need for role differentiation:

“Existential questions are part of being human, but they are not something you can talk about easily, it requires the support of a spiritual counsellor. A care worker can learn to give attention, by asking questions and so on” (R1, A).

#### 3.2.2. Personal communication and relational requirements

As a competence of health care personnel, the documents on interviews with personnel show that they are expected to be able to recognize existential questions. Above all, care personnel are expected to communicate well, and to combine this with the use of tools such as recording existential questions in the care plan. Communicating well implies listening well, not just to what is actually said but also to what is communicated “between the lines”. An ability to deal with conflicting interests of the resident and his/her family also emerges as a desirable competence in this context. Additionally, it is stated to be important that the personnel are able to create the right conditions: to schedule talks with residents; to reserve time, if necessary to refer a resident to experts; to be sufficiently aware of the resident’s personal history and interests. The documents show that these competences require structural conditions on the part of the organization, such as sufficient continuity in the personnel to enable the building of trust, and enough time for the personnel to devote the necessary attention to residents.

Specifying some requirements put forward above, the in-depth interviews and participant observations indicate a number of requirements that start from the residents’ personal relationships with personnel and that focus on the roles of various actors. As regards the conversation skills of care personnel, residents wish they were able to enquire further into certain matters, so that the contact can grow beyond superficial contact. This requires that care) workers are alert to signals and take the initiative. However, residents also say that the personnel should have the sensitivity to notice whether a resident is eager to talk or would rather be left alone. In other words: attention for existential questions also means giving the opportunity *not* to talk about them or to otherwise deal with them. The participant observations confirmed residents’ wish for a balance between alleviating loneliness by giving attention and leaving alone those who wish to be left alone. In other words, the personnel need to be responsive to the residents’ personal experience and needs, where possible. Personnel are expected to build a bond of trust with the residents, allowing them to engage the residents in conversation in a casual manner, yet in such a way that they do have “real contact”. What matters is to create space where residents can talk about more profound things.

In this respect, interviewees also mention simple social interaction and conviviality as important aspects, where one doesn’t need to talk about “heavy issues”. On the other hand, residents also mention that personnel should be able to address negative matters. Residents do wish for some personal contact, but personnel should not burden residents with their own personal issues.

A further requirement for personnel that residents desire is that personnel are aware of the resident’s contact with his/her own social network. A number of residents describe how important the nearness and practical support of their children is to them. One resident indicates that she prefers to discuss deeper personal matters with them than with the personnel. Yet many residents also find it difficult to talk about existential questions with their children, because they do not want to burden them with these or because they are not accustomed to discussing such matters with them.

#### 3.2.3. Conclusions

The documents, interviews and participant observations show that dealing with existential questions puts clear demands on the care organization and its care personnel. It is indicated that the organization should create the necessary conditions, such as time, space and casual contact moments. It is important for personnel to be aware of each other’s presence and role: who does what, who is the spiritual counsellor, and when can or should a resident be referred, and then to whom? The different roles and positions should also be clear for the residents. When a care worker is in contact with a resident, it is important for him/her to listen closely, to probe further and to be responsive to the resident’s need. Residents appreciate being asked regularly how they are doing, and then to talk a bit further. One important demand formulated for the spiritual counsellor—aside from creating clarity on his/her role and position—is that he/she actively visits residents and invites them to participate in church or other contemplative meetings and discussion groups. With respect to residents’ own social network, it is important for the personnel to be aware of the resident’s contact with that network.

## 4. Conclusions and discussion

### 4.1. Conclusions

This research was set up as a qualitative case study to obtain insight into how organizations for long-term elderly care deal with existential questions and what kind of requirements are needed for dealing well with them. We used theoretical notions on ultimate concerns, life events and meaning in life as input to our empirical explorations. The combination of interviews, participant observations and document study produced a broad picture of how existential questions are dealt with.

It emerged that both long-term care organizations had hardly any explicit and concrete policy on dealing with existential questions, while they were involved in dealing with “existential questions” and active in the related pilot programme. Whatever policy and activities do exist on this point is often not very familiar to the personnel, and the approach is generally not systematic. The personnel have few specific tools to deal with existential questions, and the lack of time means that they are often unsure of what to do, except to try to lend a sympathetic ear. It can be assumed that problems in dealing with existential questions will be even greater in long-term care organizations that are less involved with policy on these issues.

As an advice personnel and residents put forward that organization policy on existential questions should be very explicitly communicated and that personnel and residents—each in their own way—should be actively supported in dealing with issues of existential questions by providing trainings and activities. Also, results showed that further demands pertain to the creation of organizational framework conditions such as the availability of a spiritual counsellor, having certain facilities in the building, and clear definitions of the various roles and positions of the personnel (also towards the residents). For the health care worker, dealing with existential question mainly requires a broad social-communicative competence.

### 4.2. Discussion

In order to explore what the main findings of this study may mean for care practices, we discuss them with a view to formulating recommendations for a professional quality framework for elderly care institutions. The discussion focuses on three main outcomes of this study.

#### 4.2.1. The resident at the centre

First, an important outcome concerned putting the resident and his/her needs at centre stage. As the documents and attended work meetings revealed, attention for existential questions often means that staff members are sensitive to “the question behind the question”: that they realize that a question can contain multiple layers of meaning. In this respect, residents find it important that personnel not only ask how they are doing, but that they also probe further. An important addition here is what Leget et al. (2010, p. 6) referred to as presence:

a manner of acting that stresses being attentively present, where support is not offered from outside, but an attempt is made to mobilize the patient’s own powers through presence, loyalty, and by letting be rather than doing (a “letting-be mode of action”).

This is not the type of approach that most care personnel are accustomed to: they are generally short of time and only pass by briefly to perform specific care treatments. That is why it is important for a quality framework, and for competence building, to complement the repertoire of actively responding to existential questions with attention for this “letting-be mode of action”.

#### 4.2.2. Competence building

Second, the documents, interviews and participant observations all reveal that staff members and volunteers feel a need for, and would be supported by, further training and peer supervision. It is of high importance to the residents’ well-being, as also shown in extra care situations (Shaw, West, Hagger, & Holland, 2016), that care givers are able to organize proper activities and can provide emotional and social support. The organization’s spiritual counsellor could play an important role in competence building here. He or she could organize and support the (peer) supervision based on his/her own experience with and knowledge of existential themes. This would simultaneously increase his/her own visibility within the organization, which may increase or facilitate referrals. It is important for a quality framework to devote attention to competence building in a broad sense: both in terms of responding to the resident and in terms of dealing with colleagues and one’s own existential questions.

#### 4.2.3. A “tiered system” for dealing with existential questions

Third, as an overarching conclusion our study showed that dealing with existential questions is relevant at multiple levels in the organization. Therefore a “tiered system” for dealing with existential questions, in which every staff department or every organizational domain can have its own role or task, seems appropriate, This recommendation is in line with the position of the Dutch national guideline on spiritual care (Leget et al., 2010; Geer & Leget, 2012, pp. 103–104) that every discipline has and should have its own working method: “Every discipline also has its own discipline-specific method of offering support: its own repertoire and role with respect to the patient.” Differentiation is essential, which is why the said guideline distinguishes between three types of situations (Leget et al., 2010, p. 16; Geer & Leget, 2012, pp. 103–104):

(A) situations in which it suffices to have everyday *attention* for existential questions in the care sector, (B) situations in which patients feel the need for *support* in the domain of existential questions, or in which they experience a normal existential struggle and the support of an expert can have added value, and (C) situations in which the existential struggle leads to an existential crisis requiring a *crisis intervention* by a spiritual counsellor, medical-social worker or psychologist.

Each distinct situation requires a tailored response to offer good care.

In addition, we found that a tailored response to existential questions also is contingent on the organizational framework conditions. These include physical facilities, the atmosphere and possibilities to implement suitable activities (like celebrations to mark important life events). This corresponds to recommendations in other studies about more attentiveness to the quality of health care environments and facilities (Shaw, West, Hagger, & Holland, 2016; Todres, Galvin, & Holloway, 2009). It is important to identify such framework conditions as a separate category, to avoid taking environmental factors for granted and hence not using these as a means to support the attention for existential questions.

If a care organization aims to deal well with existential questions, what does a tiered system then mean for the various staff members? Our study suggests differentiation in tasks and responsibilities for five categories of staff members:

1. Management and policy workers: creating conditions to facilitate attention for existential questions. This may include financial conditions, the furnishing of the building, hiring the right personnel (including a spiritual counsellor), training the personnel, organizing structural activities, and so on.
2. Spiritual counsellors: talking to residents (and personnel) about existential questions, contributing to a humanisation of the organization, in part by offering trainings and competence building;
3. Care personnel: learning to recognize existential questions, developing conversation skills, referring residents to the spiritual counsellor;
4. Facilities staff: contributing to a good atmosphere and pleasant environment in which there is room for existential questions, referring residents to the care personnel;
5. Volunteers: being competent in conversation skills, offering residents easy and open contact, referring residents to the care personnel.

Precisely because of the differentiation in roles, dealing with existential questions shows up as a shared responsibility within the care organization.

### 4.3. Final remarks

Dealing with existential questions in elderly care is a relatively new field, in both theoretical and practical respects. Given the ageing population in many countries, it is moreover a highly relevant field, requiring further scientific research and practical guidelines. Developing a quality framework for elderly people in long-term care is complicated, certainly if it is meant to reflect and support elderly people’s everyday responses to existential questions. By means of an open and exploratory approach, this study aimed to provide insight in how long-term care organizations deal with existential questions, what kind of requirements may be necessary, and what this may mean for a professional quality framework. More qualitative studies are needed, in particular on how the quality of dealing with existential questions can be established. The qualitative outcomes may also lead to the advance of quantitative measurement instruments that fit the substantive aims of a quality framework. Of principal importance is to develop research methods that adequately relate to this population of vulnerable older people, their specific issues on existential questions, and the different disciplines and organizational domains involved in long-term elderly care.
